# Prevention of shoulder injuries in overhead athletes: a science-based approach

**DOI:** 10.1590/bjpt-rbf.2014.0109

**Published:** 2015-09-01

**Authors:** Ann M. Cools, Fredrik R. Johansson, Dorien Borms, Annelies Maenhout

**Affiliations:** 1 Department of Rehabilitation Sciences and Physiotherapy, Faculty of Medicine and Health Sciences, University Hospital Ghent, Ghent, Belgium

**Keywords:** shoulder, injury prevention, return to play

## Abstract

The shoulder is at high risk for injury during overhead sports, in particular in throwing or hitting activities, such as baseball, tennis, handball, and volleyball. In order to create a scientific basis for the prevention of recurrent injuries in overhead athletes, four steps need to be undertaken: (1) risk factors for injury and re-injury need to be defined; (2) established risk factors may be used as return-to-play criteria, with cut-off values based on normative databases; (3) these variables need to be measured using reliable, valid assessment tools and procedures; and (4) preventative training programs need to be designed and implemented into the training program of the athlete in order to prevent re-injury. In general, three risk factors have been defined that may form the basis for recommendations for the prevention of recurrent injury and return to play after injury: glenohumeral internal-rotation deficit (GIRD); rotator cuff strength, in particular the strength of the external rotators; and scapular dyskinesis, in particular scapular position and strength.

## Introduction

The shoulder is at high risk of injury in overhead sports like tennis or volleyball because it faces high loads and forces during serving and smashing. Injury risk seems to increase with age^1^^,^ 2 and, despite some lack of evidence, has been suggested to be related to level and volume of play^2^^-^ 4.

Most of the reported shoulder injuries are strains, implicating a process over time, with chronic overload leading to injury^1^. Chronic shoulder pain in the overhead athlete is often attributed to sport-specific adaptations, alterations in strength, flexibility, and posture not only in the glenohumeral joint, but also in other links of the kinetic chain^5-9^. These alterations change biomechanics and movement strategies during serving and striking, possibly leading to overload injuries at the shoulder. In particular, glenohumeral internal-rotation deficit (GIRD), rotator cuff strength imbalance, scapular dyskinesis, thoracic spine stiffness and hyperkyphosis, lumbar core instability, and hip range of motion and strength deficits possibly create the "cascade to injury", as defined by Kibler^10^ and Lintner et al.^7^ in overhead athletes. This kinetic chain "breakage" has been suggested to be a result of repetitive, vigorous activities in both young and older athletes^7^^,^ 10 ^,^ 11. In spite of the relevance of kinetic chain alterations in the spine and lower extremities, the discussion of these variables is beyond the scope of this paper, which focusses on more local shoulder girdle factors.

In order to create a scientific basis for the prevention of recurrent injuries in overhead athletes, four steps need to be undertaken: (1) risk factors for injury and re-injury need to be defined^12^; (2) established risk factors may be used as return-to-play criteria, with cut-off values based on normative databases; (3) these variables need to be measured using reliable, valid assessment tools and procedures; and (4) preventive training programs need to be designed and implemented into the training program of the athlete in order to prevent re-injury. The purpose of the present paper is to review the literature regarding these steps and to suggest some clinical applications of the current knowledge to the clinician.

## Risk factors for shoulder injury in overhead athletes

In spite of promising results from prospective studies, no consensus exists regarding intrinsic risk factors for shoulder pain in the overhead athlete. Different requirements on the shoulder and specific throwing activities across the spectrum of overhead athletes might account for these discrepancies. Recently GIRD and rotator cuff strength deficit, as well as scapular dyskinesis have been defined as possible risk factors in a population of baseball, rugby, and handball players^13^^-^ 18. In particular, pre-season reduced internal rotation range of motion^14^, reduced total range of motion^13^^,^ 15, a strength deficit in the external rotators^13^^,^ 16 ^,^ 17, and inadequate scapular position during clinical testing^13^^,^ 18 were shown to increase the risk for overuse chronic shoulder pain in these athletes.

Posterior shoulder stiffness is a common, if not the most common, adaptation seen on the dominant side of overhead athletes of multiple sports disciplines^8^. This manifests clinically as decreased glenohumeral cross-body adduction and internal rotation mobility and is believed to be the result of both capsular tightness and muscular contracture. It is hypothesized that the cumulative loads onto the posterior shoulder during the deceleration phase of the throwing motion cause microtrauma and scarring of these soft tissues^8^. Posterior shoulder stiffness, therefore, has been suggested to be a causative or perpetuating factor in shoulder impingement and labral pathology^9^^,^ 19 ^,^ 20. Abnormal humeral head translations, caused by selective tightening of the posterior-inferior capsule, may decrease the width of the subacromial space, thus causing subacromial impingement^21^. Other studies^22^ suggest a posterior and superior translation of the humeral head during cocking with a tight posterior capsule, possibly leading to an encroachment of the rotator cuff tendons against the postero-superior rim of the glenoid. In addition, posterior shoulder tightness seems to affect kinematics of the scapula and the humeral head. and is associated with a decreased acromiohumeral distance^23^. As a result, posterior capsule shortness possibly increases the risk for internal as well as subacromial impingement in the overhead athlete^21^^,^ 22. Recently, Clarsen et al.^13^ showed an odds ratio for sports-related shoulder pain of 0.77 per 5° change in total range of motion (adding up internal and external range of motion) in a population of handball players.

During overhead throwing and serving, the shoulder is highly loaded with an enormous challenge for the eccentric capacity of the external rotators during the deceleration phase. In specific sports such as tennis, it has been shown that elite players without shoulder injury have shoulder rotation muscle strength imbalances that alter the ratio between rotator cuff muscles^24^. Although these differences do not seem to affect the athletic performance immediately, detection and prevention with exercise programs at an early age are recommended, since recently decreased external rotation strength has been identified as a risk factor for shoulder pain^13^.

There is a body of evidence showing an association between scapular dysfunction and shoulder pain, specifically in the overhead athlete^25^^-^ 30, however there is no consensus regarding the cause-consequence relationship between both clinical entities. Some studies revealed no causative relationship between scapular dysfunction and shoulder pain^31^^,^ 32, whereas others clearly identified scapular dyskinesis as a possible risk factor for chronic shoulder pain in a population of overhead athletes^13^^,^ 18 ^,^ 31. In particular, obvious scapular dyskinesis, as defined by McClure et al.^33^, and type III scapular dyskinesis, as defined by Kibler et al.^34^, were found to increase the risk for shoulder pain^13,18^. Other studies discussed scapular position in healthy tennis players, but also with conflicting results. While Silva et al.^35^ showed abnormal scapular position correlated with decreased acromiohumeral distance, Cools et al.^36^ described positive alterations in elite tennis players with increased scapular upward rotation on the dominant side.

In summary, glenohumeral range of motion, rotator cuff strength or imbalance, and scapular position and movement are important factors in the assessment of healthy and previously injured overhead athletes in order to define risk factors and guide the athlete into the return-to-play stage after injury.

In addition to the more local risk factors mentioned above, more functional deficits might be risk factors for injury like faulty biomechanics, throwing fatigue etc. In order to measure these variables, there is a need for functional testing in a throwing-specific position, for instance endurance tests of the shoulder into a throwing position, throwing distance, speed and accuracy. However, with the exception of some tests mimicking shoulder function, like the "seated medicine ball throw"^37^ or the "Y-balance test for the upper limb"^38^, to date no science-based functional test has been fully validated to determine risk factors for shoulder injury or return to play after injury.

## Return-to-play criteria based on cut-off values from the risk factors

According to the decision-based return-to-play model described by Matheson et al.^39^, 3 steps need to be taken prior to full return to sports. First, the health status of the athlete is evaluated, including assessment of symptoms and a battery of analytical and functional tests (e.g. strength and flexibility, throwing performance, etc.). Then, the clinician evaluates the participation risk based on the type of sport, level of competition, and ability to protect the shoulder. Finally, some factors might modify the decision, such as the timing in the season, pressure from the athlete, or his environment. However, in spite of this science-based model to be implemented into clinical practice, little evidence exists regarding the physical return-to-play criteria of the shoulder after injury. From a clinical perspective, there is a need for cut-off values for each of the described risk factors to be used as criteria for return to training and return to play. In addition, the clinician needs objective and valid assessment tools applicable to the athlete's field or training area. Finally, once deficits are assessed, there is a need for science-based training programs to restore normal values. The purpose of the following paragraphs is to discuss cut-off values, assessment tools, and intervention programs for GIRD, rotator cuff strength deficit, and scapular dyskinesis.

### Glenohumeral range of motion

With respect to range of motion, loss of internal range of motion is a known risk factor for chronic shoulder pain^14^^,^ 15 ^,^ 40. There is no consensus in literature with respect to the cut-off values for internal ROM, ranging from 18°^15^ up to 25°^14^ depending on the study design and population. Therefore, in view of maximal protection of the athlete, it is advised that side differences in internal rotation ROM should be less than 18°, and the difference in total range of motion should be no more than 5°^15^. The studies referring to selectively measuring GIRD, base their instruction on the proposition that it is the result of selective tightening of the posterior shoulder structures, such as the posterior capsule of the glenohumeral joint and the posterior cuff muscles. The relevance of the concept of total range of motion, in which internal and external ROM are added up, has been introduced in the literature since the first studies showing bony adaptations in the humeral torsion based on overhead sports activity^41^. Increased humeral torsion alters the arc of total range of motion into decreased internal rotation ROM and increased external rotation ROM. In this hypothesis, the athlete is not at risk as long as the loss of IR is compensated by a gain of ER. Therefore, it is advised, in particular in elite athletes, to take into account the total ROM rather than the internal rotation ROM as a risk factor. A recent study on professional baseball players found that pitchers with GIRD displayed greater side-to-side differences and dominant humeral retrotorsion compared to those without GIRD. The authors concluded that the greater humeral retrotorsion may place greater stress on the posterior shoulder resulting in ROM deficits. Pitchers with greater humeral retrotorsion appear to be more susceptible to developing ROM deficits associated with injury and may need increased monitoring and customized treatment programs to mitigate their increased injury risk^42^.

**Figure 1.**
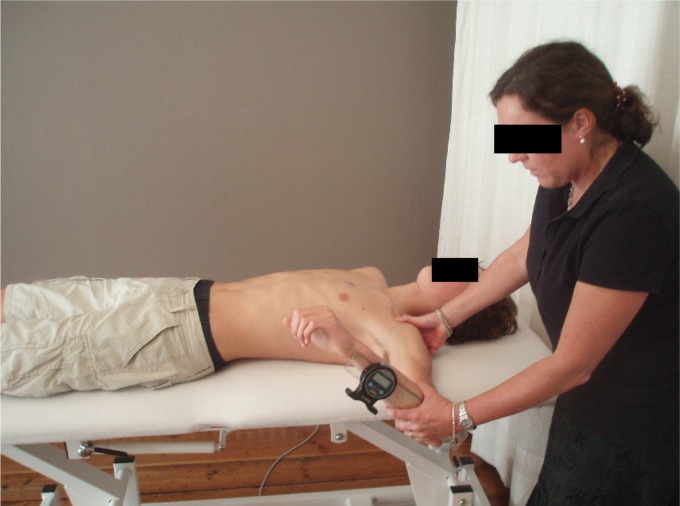
Measurement of internal rotation of the shoulder using a digital inclinometer^24^.

The assessment of the ROM into rotation of the shoulder can be measured with a goniometer or an inclinometer, and in many positions of the body and the shoulder. A comprehensive reliability study^43^ showed high to excellent inter- and intra-tester reliability for a variety of test positions and equipment. Based on the results of this study, no specific procedure can be acknowledged to be superior to another one. However, the clinician has to take into account that there is great variability in the literature regarding shoulder position (e.g. scapular or frontal plane)^15^^,^ 24 and the specific method of scapular stabilization (none, hand on shoulder top, or specific fixation of coracoid). Based on the above-mentioned reliability study and in view of optimal standardization of body and shoulder position, the authors advise the following procedure: the patient is supine with the shoulder in the frontal plane and the elbow flexed 90°. The upper arm should be horizontal or if needed the arm can be supported by a towel to reach the horizontal position (for instance in case the patient has protracted shoulders or a thoracic kyphosis). For internal rotation, the examiner palpates the spine of the scapula and the coracoid. The inclinometer is aligned with the forearm (olecranon and styloid process of the ulna), and the shoulder is moved into internal rotation (Figure 1). The movement reaches its endpoint when the coracoid tends to move against the palpating thumb. For external rotation, the fixating hand is placed gently over the shoulder top, and the shoulder is moved into external rotation, aligning the inclinometer with the forearm.

In addition, horizontal adduction can be measured in the assessment of posterior capsule stiffness^23^. It is advised that measurement be performed with the shoulder at 90° of flexion and horizontally adducted until the scapula starts moving laterally. While one investigator manually fixes the lateral border of the scapula and palpates the lateral movement of the scapula, the second moves the upper arm toward horizontal adduction and measures the angle between the upper arm and the vertical^23^. In spite of the clinical relevance of this measurement, its predictive value in shoulder pain is unclear.

**Figure 2.**
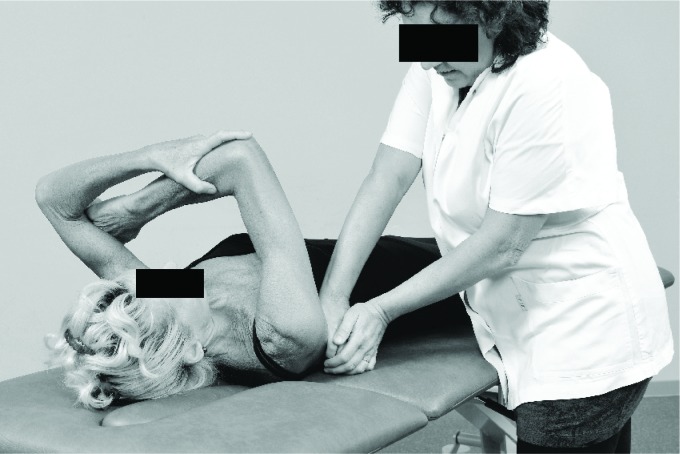
Cross body stretch^9^.

**Figure 3.**
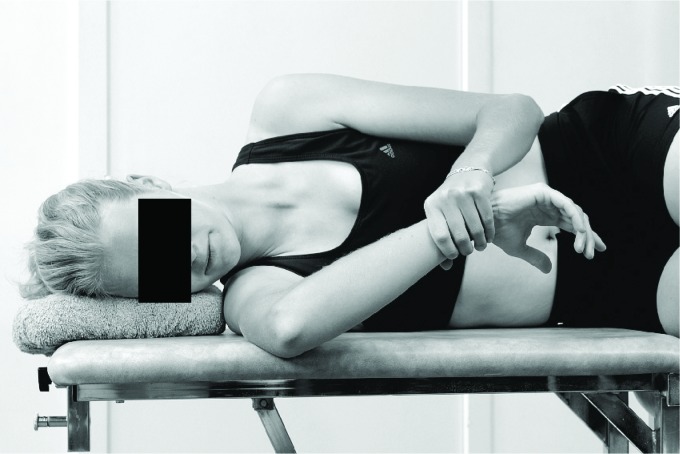
Sleeper's stretch^41^.

Given the evidenced impact of posterior shoulder tightness on shoulder kinematics, increasing posterior shoulder flexibility is advised when mobility deficits exceed the limits associated with increased injury risk. Both the cross-body stretch (Figure 2) and the sleeper stretch (Figure 3) can be recommended to decrease posterior shoulder tightness^44^. It was shown that a 6-week daily sleeper stretch program (3 reps of 30 seconds) is able to significantly increase the acromiohumeral distance in the dominant shoulder of healthy overhead athletes with GIRD^23^. Additional joint mobilization performed by a physical therapist has a small but non-significant advantage over a home stretching program alone^45^. No difference in mobility gain was seen after angular (sleeper stretch and horizontal adduction stretch) and non-angular (dorsal and caudal humeral head glides) joint mobilization by a physical therapist^46^ in a 3-week stretching program in overhead athletes with impingement-related shoulder pain. Both programs however resulted in increased ROM and decreased pain during physical examination and improved shoulder functional outcome scores. Muscle energy techniques (hold-relax) during the sleeper stretch and the horizontal adduction stretch have proven useful to immediately increase internal rotation range of motion^47^. Two studies^46^^,^ 48 showed symptom relief after a stretching program in a population of overhead athletes with impingement-related shoulder pain. However, there is no evidence to support that a stretching program reduces the incidence of recurrent shoulder injury.

### Rotator cuff strength

Regarding rotator cuff strength, it is generally recognized that overhead athletes often exhibit sport-specific adaptations leading to a relative decrease in the strength of the external rotators, and thus muscular imbalance in the rotator cuff. Isokinetic^49^ as well as isometric^24^ and eccentric^50^ strength studies have been performed in healthy and injured athletes showing deficiencies in external rotator muscle performance. In these studies, absolute side differences as well as muscle balance ratio between external and internal rotators were examined. In general, with respect to cut-off values distinguishing a healthy shoulder from a shoulder at risk, an isokinetic ER/IR ratio of 66% or an isometric ER/IR ratio of 75% is advised, with a general rotator cuff strength increase of 10% of the dominant throwing side^16^^,^ 24 ^,^ 49 compared to the non-dominant side. Recently, focus has shifted from isometric or concentric to eccentric muscle strength of the rotator cuff. In particular, the eccentric strength of the external rotators are of interest^51^. These muscles function as a decelerator mechanism during powerful throwing, serving, or smashing.

In view of the importance of eccentric rotator cuff strength in relation to injury-free overhead throwing or serving, it is imperative that strength be assessed on a regular base in healthy as well as injured players. Numerous testing protocols have been described to examine isokinetic^52^^-^ 54 and isometric^55^ rotator cuff strength. The golden standard in strength measurement is the use of isokinetic devices, however these procedures are rather expensive, and not applicable on the field or training area. With respect to the isometric strength measurements, hand-held dynamometry (HHD) has attracted more and more interest during the last years due to the more practical, less expensive and user-friendly advantages over the more advanced and expensive isokinetic devices. HHD has demonstrated higher sensitivity and intra- and inter-examiner reliability than manual muscle testing in identifying strength deficits of the rotator cuff^56^.

**Figure 4.**
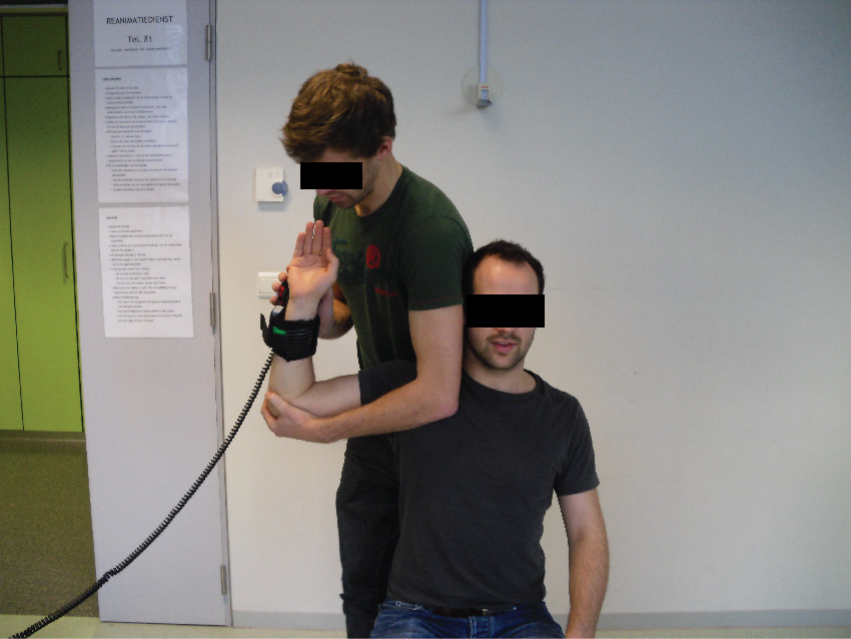
Eccentric testing protocol using an HHD^51^.

Recently, a new testing protocol was published, showing that HHD measurements of eccentric external rotator strength show excellent intra-tester (ICC=0.88) and good inter-tester (ICC=0.71) reliability, as well as concurrent validity (compared to an isokinetic device, Pearson's correlation = 0.78)^51^. During the procedure, the patient is seated gently supported by the arm of the tester, who brings the shoulder from 90° abduction-90° external rotation (throwing position) to 90° abduction-0° external rotation, loading the external rotators eccentrically (Figure 4). A large normative database on 200 overhead athletes (volleyball, tennis, and handball) was recently set up (unpublished data) and shows an average normalized eccentric external rotator strength (N/kg) of approximately 2, with significant side differences in favor of the dominant sides, and significant higher values for handball and tennis compared to volleyball.

**Figure 5.**
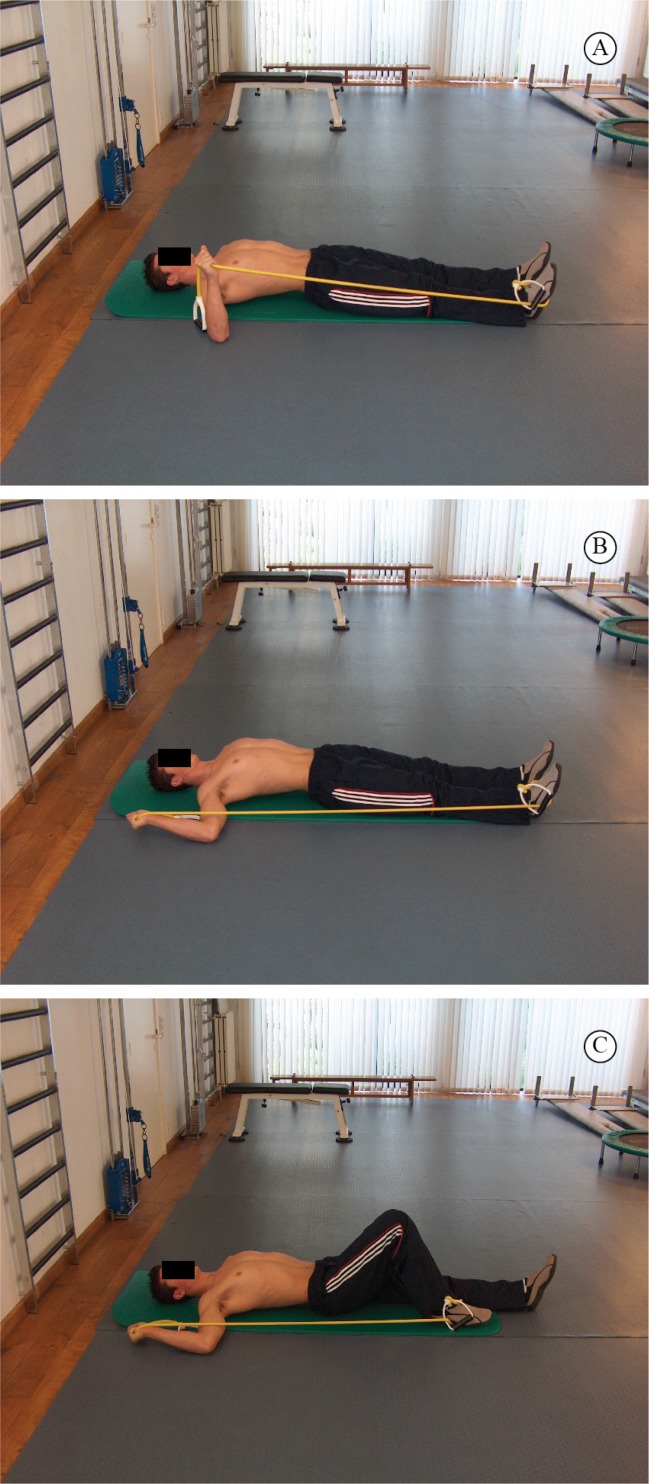
Eccentric exercise for the external rotators in an abducted position.

**Figure 6.**
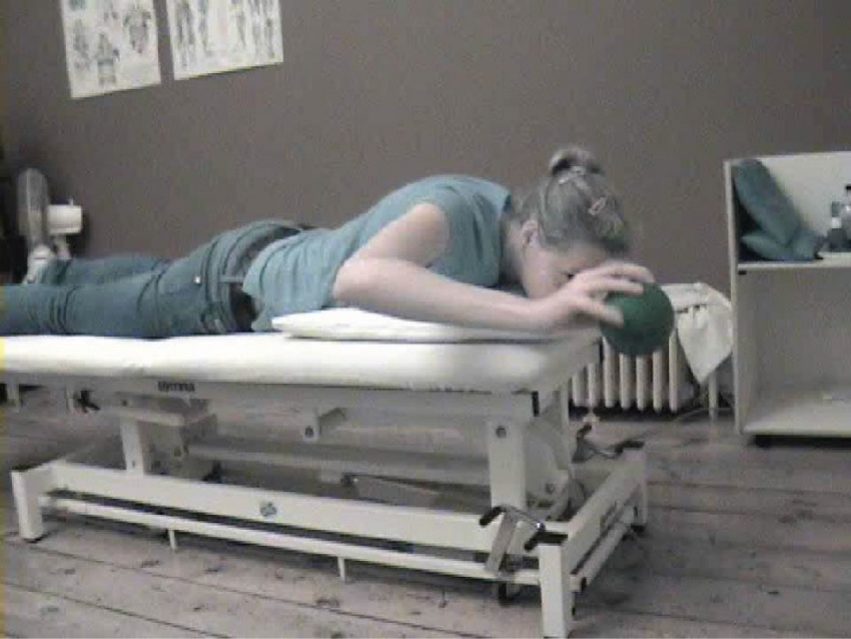
"Catching" exercise using a plyoball^41^.

**Figure 7.**
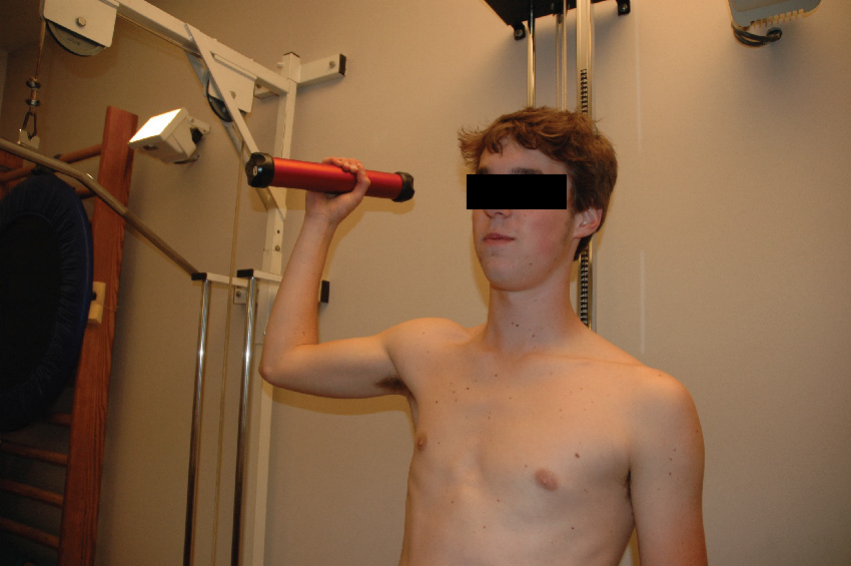
Stretch-shortening cycle exercise, using the XCO^®^trainer

Numerous exercises have been described to strengthen the rotator cuff muscles, including concentric, isometric, eccentric, and plyometric exercises^41^. In view of the eccentric component of the function of the external rotators, the sport-specific exercises for overhead athletes should focus on three areas:

1) Exercises that accentuate the eccentric phase and "avoid" the concentric phase in order to load the muscles based on their eccentric capacity. Figures 5 A-C show an example of an eccentric exercise for the external rotators in general in an abducted position.

2) Slow exercises for absolute strength, fast exercises for endurance and plyometric capacity. Endurance and plyometric capacity may be exercised using weight balls exercises in which the patient is instructed to "catch" the ball (Figure 6), as described by Ellenbecker and Cools^41^.

3) Exercise highlighting the "stretch-shortening-cycle" of throwing. Specific devices can be used to train the stretch-shortening cycle, such as XCO^®^ trainer (Figure 7).

### Scapular dyskinesis

Evidence supporting cut-off values for prevention of injury or return to play after injury with respect to scapular function is scarce. A number of studies used visual observation as a criterion^13^^,^ 18 whereas others provide objective data on healthy athletes as a reference base for return to play^36^^,^ 57. In general, visual observation is performed either by using the yes/no method (scapular dyskinesis or not), a method proven to be reliable and valid if the examiner/therapist is educated in a standardized manner^33^^,^ 58, or by categorizing the scapular dysfunction into different types, based on the specific position of the scapula^34^. However, the latter method was shown to have acceptable intra-rater, but low inter-rater reliability^34^. Clarsen et al.^13^ rated scapular dyskinesis in handball players as having normal scapular control, slight scapular dyskinesis, or obvious dyskinesis^13^, and established obvious dyskinesis as a risk factor for shoulder pain. A statement saying that scapular behavior should be symmetrical in overhead athletes is not supported by research data. On the contrary, in volleyball as well as in handball players, asymmetry was found in resting scapular posture^57^^,^ 59. Uhl et al.^58^ also reported that the prevalence of scapular dyskinesis was almost identical in subjects with and without shoulder pain, questioning the clinical value of scapular asymmetry. Therefore, clinicians should be aware that some degree of scapular asymmetry may be normal in some athletes. It should not be considered automatically as a pathological sign, but rather an adaptation to sports practice and extensive use of upper limb.

Several studies measured scapular upward inclination in healthy overhead athletes^46^^,^ 60. These data may be used as a reference base and cut-off values for correct scapular positioning in several elevation angles. In general, a large variety is found in scapular upward inclination in the midrange of motion (probably due to a large variation between individuals), however in full elevation, most studies suggest that upward inclination should be at least 45-55°^36^^,^ 60.

For the scapular muscles proper inter- and intramuscular balance should be assessed. Isokinetic ratio protraction/retraction is shown to be 100% in a healthy population, with slight changes in overhead athletes, in case of throwing athletes in favor of the protractors^25^^,^ 36 ^,^ 61. In bilateral sports (swimming, rowing, gymnastics), there should be no side differences in scapular muscle strength. In one-handed overhead sports, an increase of 10% in scapular muscle strength is advised on the dominant side. In particular, the lower trapezius and serratus anterior should receive special attention, since these muscles are shown to be susceptible to weakness in injured athletes^10^^,^ 62.

In the assessment of scapular behavior, besides the clinical observation, several measurements can be performed for scapular position as well as muscle strength. The use of a digital inclinometer for the measurement of scapular upward rotation has been shown to exhibit high inter- and intra-rater reliability^60^. Key conditions for good measurements are adequate palpation of the reference points in the different humeral elevation angles and control of additional tilting of the inclinometer in planes other than the scapular plane. For the measurement of scapular muscle strength, several protocols have been described^36^^,^ 63. Differences between procedures are based on the equipment used, positioning of the dynamometer, patient positioning, and performing a "make" and "break" test. Different testing procedures result in different outcome, the clinician should take that into account using reference data from research in the clinical practice. In the authors' experience, using the Kendall & Kendall position and performing a "make" test with a hand held dynamometer is an acceptable and clinically relevant method of strength measurement of the scapular muscles^36^.

Once deficits and imbalances in scapular behavior are assessed, an intervention program to restore flexibility and muscle performance needs to be installed. Recently, a science-based clinical reasoning algorithm was published guiding the clinician into the different steps and progression^62^. The main goals are: a) to restore flexibility of the surrounding soft tissue of the scapula, in particular pectoralis minor, levator scapulae, rhomboid, and posterior shoulder structures; and b) to increase scapular muscle performance around the scapula, focusing on either muscle control and inter- and intramuscular coordination or muscle strength and balance. Exercises to restore scapular muscle balance^64^ have been shown to increase isokinetic protraction and retraction^65^, increase external rotator strength of the shoulder^66^, and alter EMG activity of the scapular muscles in favor of efficient muscle recruitment during a loaded elevation task^67^.

## Conclusion

In summary, with respect to injury prevention as well as return to play after injury, the clinician should evaluate possible risk factors for injury in the shoulder, in particular GIRD, rotator cuff strength, and scapular performance, using reliable assessment tools. In case abnormal findings are established, the intervention should focus on stretching of the posterior shoulder capsule, strengthening of the posterior cuff, and restoration of flexibility and muscle balance of the scapular muscles.
